# Circulating tumor cell markers for early detection and drug resistance assessment through liquid biopsy

**DOI:** 10.3389/fonc.2025.1494723

**Published:** 2025-04-07

**Authors:** Priya Yadav, Saravanan Rajendrasozhan, Ramzi Hadj Lajimi, Raja Ramadevi Patel, Dominique Heymann, N. Rajendra Prasad

**Affiliations:** ^1^ Department of Biochemistry and Biotechnology, Annamalai University, Chidambaram, Tamil Nadu, India; ^2^ Department of Chemistry, College of Science, University of Ha’il, Ha’il, Saudi Arabia; ^3^ Medical and Diagnostic Research Centre, University of Ha’il, Ha’il, Saudi Arabia; ^4^ Department of Biology, College of Science, University of Ha’il, Ha’il, Saudi Arabia; ^5^ Nantes Université, CNRS, US2B, UMR 6286, Nantes, France; ^6^ Institut de Cancérologie de l’Ouest, Tumor Heterogeneity and Precision Medecine Laboratory, Saint-Herblain, France; ^7^ Medical School, University of Sheffield, Sheffield, United Kingdom

**Keywords:** liquid biopsies, circulating tumor cells, multidrug resistance, tumor plasticity, early detection, nanotechnology

## Abstract

Circulating tumor cells (CTCs) are cancerous cells that extravasate from the primary tumor or metastatic foci and travel through the bloodstream to distant organs. CTCs provide crucial insights into cancer metastasis, the evolution of tumor genotypes during treatment, and the development of chemo- and/or radio-resistance during disease progression. The process of Epithelial-to-mesenchymal transition (EMT) plays a key role in CTCs formation, as this process enhances cell’s migration properties and is often associated with increased invasiveness thereby leading to chemotherapy resistance. During the EMT process, tumor cells lose epithelial markers like EpCAM and acquire mesenchymal markers such as vimentin driven by transcription factors like Snail and Twist. CTCs are typically identified using specific cell surface markers, which vary depending on the cancer type. Common markers include EpCAM, used for epithelial cancers; CD44 and CD24, which are associated with cancer stem cells; and cytokeratins, such as CK8 and CK18. Other markers like HER2/neu and vimentin can also be used to target CTCs in specific cancer types and stages. Commonly, immune-based isolation techniques are being implemented for the isolation and enrichment of CTCs. This review emphasizes the clinical relevance of CTCs, particularly in understanding drug resistance mechanisms, and underscores the importance of EMT-derived CTCs in multidrug resistance (MDR). Moreover, the review also discusses CTCs-specific surface markers that are crucial for their isolation and enrichment. Ultimately, the EMT-specific markers found in CTCs could provide significant information to halt the disease progression and enable personalized therapies.

## 1 Introduction

Cancer is a rarely curable disease and has an increasing overall burden in the world. According to the National Center for Health Statistics, USA estimated about 1.95 million new cancer cases and 0.6 million cancer-related deaths were reported in 2023 (1). Therapeutic options for different types of cancers often include local and/or systematic approaches. The local treatments include radiation and surgery whereas systematic treatment comprises chemotherapy and targeted therapy such as immunotherapy (2). Most existing cancer chemotherapy undergoes failure in almost 90% of cases mainly due to the development of resistance against the administered therapeutic agents (3). Drug resistance is a milestone in disease progression, often resulting in accelerated disease progression, leading to the emergence of resistant clones that may exhibit aggressive characteristics, ultimately contributing to overall treatment failure. In this context, due to the development of drug resistance, 50% of patients with non-small cell lung carcinoma (NSCLS) advanced stage, and around 70% of ovarian adenocarcinoma (stage 1) patients experience a relapse during the therapies followed by death (4, 5), and about 20% of pediatric acute lymphoblastic leukemia (stage 1) cases are associated with recurrence episodes (6). Similarly, approximately 50% of women suffering from HER2-positive (HER2^+ve^) breast cancer (early stages) acquire resistance to trastuzumab (7). Consequently, the risk profile of death in cancer patients is 36% within the first 5 years of recurrence (8). In the current scenario, the foremost priority of researchers and clinicians is to set up (1) an ultrasensitive diagnostic method that detects the cancer in its early stage; and (2) effective treatment to overcome drug resistance and adverse toxic reactions in the non-targeted organs. Circulating tumor cells (CTCs) express several drug resistance markers, including drug efflux pumps which actively pump out the drugs and reduce their effectiveness. Further, markers like HER2/neu and EGFR can contribute to resistance through overexpression or mutations. This review emphasizes that the investigation of CTC is an ultrasensitive diagnostic method for early detection of cancer. Additionally, identifying the specific drug resistance markers expressed by CTCs such as drug efflux pumps, HER2/neu, and EGFR helps to develop therapeutic strategies that can effectively overcome resistance thereby minimizing adverse effects on non-targeted organs. Furthermore, this facilitates improved treatment outcomes and reduces the risk of cancer recurrence in patients.

## 2 Cancer detection and monitoring

Cancer detection typically begins with non-invasive imaging techniques such as medical imaging and microscopic analysis to identify the presence of tumors. High-resolution image-based techniques are valuable for initial tumor detection but also have limitations such as lack of sensitivity and can provide unclear or delayed data which may hinder timely diagnosis and therapy (9). Furthermore, these imaging-based techniques are unable to detect early-stage tumor cells, which can delay intervention. Once a tumor is detected diagnosis is often confirmed through tissue biopsy with needle biopsies being the most commonly used method for investigating cancer pathologies and characterizing its molecular features (10). However, tissue biopsies have several limitations including they are invasive procedure that provides no information about the metastatic status of cancer and some tumor sites are not accessible for biopsy. Additionally, biopsies can lead to complications such as infections, heavy bleeding, and discomfort for patients. For example, endometrial biopsies may result in pelvic pain, bleeding, and infection (11), while bone cancer biopsies can be of poor quality and may require a decalcification approach that may compromise the molecular analysis (12). Furthermore, tumors attain heterogeneity, and tissue biopsy samples at a specific time point and location may not accurately represent the tumor’s overall molecular landscape. This is particularly problematic in advanced cancers where tumor re-biopsy is often not feasible due to ethical concerns and the high risk of infection (13). Therefore, using tissue biopsies to monitor treatment response and detect relapse throughout therapy presents significant challenges especially given that tumors can evolve rapidly under therapeutic pressure. To overcome the existing drawbacks of imaging based techniques and tissue biopsies, liquid biopsies have emerged as a promising alternative for cancer diagnosis. Liquid biopsies offer a less invasive and provide insights into characterizing their molecular features and monitoring disease progression. They are particularly useful for assessing tumor prognosis, detecting cancers at both early and advanced stages, and tracking responses to treatment. Moreover, liquid biopsies can be employed to identify recurrences and monitor the emergence of drug resistance, making them a valuable tool for ongoing cancer management.

## 3 Liquid biopsy and its significance

Liquid biopsies are techniques that involve the collection of body fluids such as saliva, blood, urine, and cerebrospinal fluid for the assessment of tumor presence and characteristics (14). These biopsies encompass a range of tumor-derived entities including circulating tumor cells (CTCs), cell-free circulating tumor DNA (ctDNA), extracellular vesicles (EVs), tumor-educated platelets (TEPs), soluble proteins, and metabolites, all of which can provide insights into tumor biology at various stages of the disease. Thus, a liquid biopsy serves as a temporal snapshot of the cancer state (15, 16). CTCs in particular, are more critical components of liquid biopsies as they are found in the peripheral blood of patients with solid tumors (17). CTCs play a significant role in metastasis by facilitating the colonization of distant sites. CTCs can be shed from both primary and metastatic tumors highlighting their potential as biomarkers for early cancer detection (18, 19). Various transmembrane glycoproteins such as epithelial cell adhesion molecule (EpCAM) (20), vimentin (21), human mucin-1 (MUC-1) (22), human epidermal growth factor receptor-2 (HER-2) (23), ephrin receptor (ephB4) (24), E-cadherin (18), N-cadherin (25), glypican-3 (GPC3) (26), trophoblast cell surface antigen (Trop-2) (27), and prostate-specific antigen (PSA) (28) are overexpressed on CTC surfaces while being absent in normal peripheral blood cells. In addition to the markers previously mentioned, CTCs can also express programmed death-ligand 1 (PD-L1), a protein that plays a critical role in the immune evasion of tumors (29). The expression of all the surface markers on CTCs is influenced by tumor type and EMT status which can affect the tumor’s aggressiveness and metastatic potential. The detection of these surface markers enhances the sensitivity of liquid biopsies for both early and advanced cancers (30). Research has revealed that CTCs that originate from primary and metastatic sites can provide a lineage tracing of tumor cell dissemination, contributing to an understanding of tumor heterogeneity. Autopsy studies in prostate cancer patients have illustrated the spread of cancer cells from primary sites to metastatic locations revealing the complexity of tumor evolution (31, 32). By reconstituting the phylogenic trees of spreading cells, the authors demonstrated the emergence of clones from one site to another which led to the increase in tumor heterogeneity. CTCs are directly linked to this autoamplication process along the therapeutic lines and provide overall information regarding the cancer (33). Therefore, the detection of circulating analytes is closely linked to therapeutic outcomes as they provide comprehensive insights into the cancer’s progression.

Among all body fluids, blood is the most relevant body fluid for liquid biopsy due to its proximity to various tumor subtypes. The quantification of CTCs in blood directly correlates with tumor progression. Li et al. demonstrated a direct relationship between CTC counts and disease progression in patients with gastric cancer (34). Liquid biopsies afford significant advantages over traditional tissue sampling techniques enabling real-time monitoring of cancer status and treatment response (33). Furthermore, molecular profiling through liquid biopsies offers insights into personalized cancer therapies, drug resistance mechanisms, and tumor relapse dynamics. Tumor heterogeneity and drug resistance are interlinked mechanisms and can originate from the presence of multiple subclones within individual tumors exhibiting distinct resistance traits (35). Key genes implicated in multidrug resistance include those encoding for drug efflux transporters, cyclin-dependent kinases, and cytochrome P450 enzymes (36). Thus, liquid biopsies represent a clinically significant approach for non-invasive tumor sampling, real-time disease monitoring, and the identification of potential biomarkers related to drug resistance in cancer patients.

## 4 Approaches for the detection of CTCs

CTCs are very important prognostic biomarkers in *liquid biopsy* for early detection of cancers and response to given systemic therapies. Despite that, the isolation and detection of CTCs from peripheral blood are often associated with challenges due to their low abundance in peripheral blood. CTCs heterogeneity is an additional obstacle due to significant variations in their surface markers. Different methods can be employed for the successful isolation and detection of CTCs based on their physical and biological characteristics. For example, protein-based biomarkers are over-expressed on the surface of CTCs that could be the target to enrich, capture, and isolate CTCs from blood (30). One such method is the Isolation by Size of Epithelial Tumor cells (ISET) approach which captures CTCs based on their size and deformability by filtering blood through a microfiltration device that selectively retains larger epithelial cells while allowing smaller cells to pass through (37). The CTCs can also be isolated efficiently based on their metabolism specifically the “Warburg effect”. Tumor cells are highly proliferative cells and need energy for cell division thereby having higher glycolysis pathways, lactate deposition, and acidic intracellular tumor microenvironment. Microfluidic techniques used for the isolation of CTCs having higher lactate deposition and secretion are another alternative, sensitive, and reliable platform for CTCs enrichment (38). However, the CTCs have wider applications in early cancer detection but suffer shortcomings in the areas of isolation and detection. Due to unique technical challenges, the isolation of CTCs from the enormous pool of blood cells is a tedious procedure. Moreover, conventional techniques for cancer diagnosis and detection are time-consuming, expensive, and need to be repeated a couple of times at least to ensure accuracy. In the era of personalized medicine, CTCs have been popularly used for the monitoring of cancer progression and the selection of appropriate chemotherapeutic regimens. The development of advanced techniques that diagnose cancer more sensitively helps to avoid the delay in cancer detection. The comprehensive insights of CTCs at the molecular level facilitate the development of new devices and technologies for their isolation and detection.

Nanotechnology could offer a platform for the efficient isolation and detection of CTCs from patients’ blood even at lower volumes (39). A wide range of nanomaterials has been developed and screened to capture the CTCs from peripheral blood. Moreover, the role of different types of nanomaterials can be widely perceived in the detection, diagnosis, and treatment of several sub-types of cancer. Different nanomaterials such as magnetic nanoparticles (40, 41), gold nanomaterials (42, 43), graphene oxide nanoparticles (43, 44), carbon-based nanomaterials (45), nanopillars (46), nanofibers (47), and nano-roughened surfaces are commonly employed at varying degrees of design sophistication. Nanomaterials used for the efficient isolation and detection of CTCs are synthesized using various methods each suited to their unique properties and applications. Magnetic nanoparticles are typically synthesized through co-precipitation where iron salts are usually mixed with a base to form nanoscale magnetic particles that can be functionalized for CTC capture via magnetic separation. Gold nanomaterials are generally synthesized by chemical reduction method and reduce gold salts to nanoparticulated forms which can further functionalized with antibodies for targeted CTC isolation. Graphene oxide is specifically synthesized by the hummers’ method where graphite is oxidized in an acidic solution, resulting in highly dispersible particles with functional groups for CTC binding. Carbon-based nanomaterials such as carbon nanotubes are generally synthesized using chemical vapor deposition providing high-quality nanomaterials for electrochemical sensing of CTCs. Nanomaterials are utilized to embed with microfluidics devices to detect the CTCs even at low sample volume in a more efficient and apace manner. Additionally, nano-enabled microfluidic devices which integrate soft lithography and nanomaterials offer miniaturized high-throughput solution for CTC isolation. The immunoaffinity-based methods and nanostructured microfluidic chips are most frequently employed for efficient isolation and enrichment of CTCs. The integration of conventional approaches to isolate and detect CTCs with nanotechnology improves its sensitivity and accuracy. Moreover, the emphasis will be given to the significance of materials used for fabricating nano-enabled devices and technologies for the detection of CTCs. Therefore, the above-mentioned diverse synthesis methods contribute to the development of nano-enabled technologies that improve CTC capture, detection, and early cancer diagnosis by enhancing the sensitivity and efficiency of isolation techniques.

Technologies have been developed consistently for CTCs isolation/detection since the early 2000s. Some of those technologies are now available as products in the market. Some commercially available techniques and devices are available for the enrichment and capturing of different types of CTCs. **Table 1** illustrates the different commercially available technologies for the isolation and capturing of CTCs. Almost all of them are highly reproducible, easy to use, and high-performance. Both label-dependent and label-independent types of isolation/detection devices are available commercially. More efforts are being made to increase performance by developing advanced microfluidic technologies, integrating *in-silico* approaches, and much more.

**Table 1:** Commercially available techniques/Methods used for the isolation and enrichment of CTCs.

Product	Technique employed	Manufactured by	Tumor types	Prognostic relevance	Disadvantages	Ref.
CellSearch^®^	anti-EpCAM targeting immunomagnetic beads and fluorescence imaging	Menarini Silicon Biosystems Inc, USA	Metastatic breast, prostate, and colorectal cancer	Enumerate the CTCs of epithelial origin in whole blood of cancer patients	Low-level EpCAM expressing CTC are not captured by this kit	(153)
ISoFluX™	immune-magnetic capture mechanism in flow flow-controlled microfluidic system	Fluxion Biosciences, USA	Metastatic lung, colorectal, breast, prostate, pancreatic, kidney, liver, and bladder cancer	Able to isolate different tumor-generated CTCs via the multiplexing method by using multiple markers	EpCAM-negative CTCs and CTCs under EMT are not detected by this system	(154)
AdnaTest	anti-EpCAM and anti-MUC1 antibody-targeting immunomagnetic beads	AdnaGen AG, Germany	Metastatic breast, colon, and castration-resistant prostate cancer	Analyzed for tumor profiling by polymerase chain reaction (PCR) assay	Probable false positive if selection markers are expressing on cells other than CTCs and non-viable cells	(155)
MagSweeper	anti-EpCAM antibody-targeting immunomagnetic beads	Stanford University, USA	Metastatic breast cancer	CTCs captured by magnetic beads for genetic profiling	Cells undergoing EMT and CTCs devoid of EpCAM can’t be captured	(156)
MACS^®^	Anti-EpCAM targeting magnetic beads of 50 nm	Miltenyi Biotec, Germany	Metastatic breast cancer	CTCs will be captured in the column	False positive and unable to capture EMT transforming CTCs	(157)
Dynabeads™	immunomagnetic microbeads of 4.5 μm	ThermoFisher Scientific Company, USA		Anti-EpCAM based employed for both positive and negative isolation methods	Autofluorescence of beads and low-efficiency	(30)
CellMag™	ferrofluid technology using immunomagnetic separation	silicon biosystems	Metastatic Breast, prostate, and colorectal cancer	Positive selection and capturing of CTCs which are utilized for fluorescent microscopy, polymerase chain reaction (PCR), and flow cytometry analysis with single-cell sorting	Only EpCAM-specific CTCs can be captured	(156)
StraightFrom	Anti-CD326 conjugated microbead	Miltenyi Biotec	Metastatic breast cancer	detect CTCs directly from a whole blood sample	low sensitivity	(158)
DEPArray™	traps individual cells in cages formed by a non-uniform electric field; Image-based cell sorting technique	Menarini Silicon Biosystems, S.p.A., Italy	small-cell lung cancer (SCLC), metastatic breast cancer	microfluidic and microelectronic enabling precise, image-based isolation of single CTCs, which can then be analyzed by Next Generation Sequencing (NGS) methods to identify genetic mutations	differential extractions which lead to inherent loss of the sample such as bead or column-based methods	(159)
Parsortix^®^	microfluidic-based technology that captures cells based on size and deformability	ANGLE North America, Inc., King of Prussia, PA	metastatic breast cancer	Captures CTCs based on their less deformable nature and larger size compared to other blood cells; reproducibly high capture efficiency; Viable CTCs offer downstream analysis	Longer processing time; Loss of cells during processing	(160)

## 5 Multidrug resistance in cancer

Multidrug resistance (MDR) to chemotherapeutic drugs is the ability of cancer cells to survive and proliferate against several structurally and functionally different chemotherapeutic drugs (36). An in-depth understanding of the different mechanisms that underline the development of MDR in cancer subtypes triggers the identification of potential strategies for overcoming the limitations in existing therapeutic modalities (48, 49). Different genetic factors including micro RNAs (miRNAs), long noncoding RNAs (lncRNAs), and MDR genes play a significant role in the development of MDR (50). The MDR mechanism that is dependent on drug transporters is attained mainly through the efflux of chemotherapeutic drugs from the cells. Drug extrusion is facilitated in a specified manner mostly due to the overexpression of ATP-binding cassette (ABC) family-based drug transporters named multidrug resistance-related proteins (MRPs). The *MDR1* gene is also known as ATP binding cassette subfamily B Member 1; *ABCB1* encodes for P-glycoproteins (P-gp). The multidrug resistance-associated protein-1 (MRP1 or ABCC1), and breast cancer resistance proteins (BRCP or ABCG2) are crucial MDR-associated ABC transporters (51). Elevated expression of drug transporters in cancer cells is correlated with poor prognosis and response to chemotherapeutic drugs in many cancer subtypes. The recent knowledge acquired from the clinical and research outcomes indicates that multiple exposure to chemotherapeutics and radiation doses treatments significantly induced molecular and genetic alterations that facilitate the reprogramming of cells to make them chemo- and/or radio-resistance (52–54). Chemotherapy is the most effective against metastatic cancers and evidence suggests that the development of MDR usually reduces the drug absorption in the cancer cells by enhanced effluxing of drugs (55). Drug transporter-independent factors that assist in the development of MDR are changes in the tumor microenvironment, epigenetic mutations, and tumor heterogeneity (52). The heterogeneity within the tumor with different phenotypes and self-renewal capacity is also a crucial factor for the development of drug resistance (2, 56). Comprehensive genomic and molecular analysis of MDR cancer cells provides detailed insight into understanding the molecular mechanisms that result in the development of drug resistance (49).

## 6 Role of CTCs in cancer metastases and prognosis

Amintas et al. have established that CTCs and their clusters both initiate metastases while CTC clusters have been reported to have better drug resistance (57). CTCs-clusters contribute significantly to metastatic competence as compared to single CTCs (57–59). For instance, Bithi et al. highlight that doxorubicin treatment given to breast tumor patients revealed the cells within clusters of tumor cells have higher viability than their single-cell counterparts (60). Evidence has indicated that CTCs survive in the bloodstream due to stem cell-like properties then named cancer-stem cells (CSCs), escaping from immunosurveillance, and possessing epithelial and mesenchymal plasticity (18, 61–64). Even if the notion of the cancer-stem cell remains controversial in oncology, it integrates multiple aspects: i) expression of cancer stemness markers (e.g. *nanog, oct4, Sox2*) (65); ii) cells with low cycling properties leading to the notion of dormant or quiescent cells; iii) intermediate and reversible profiles of drug sensitivity highlighted by persister cells (66, 67). These properties are completed by the high plasticity of molecular profiles of CTCs that strengthen the diversity of detectable CTCs (68–71). **Table 2** demonstrates the overview of drug-resistant mechanisms in CTCs across different cancer sub-types and their impact on treatment outcomes.

**Table 2:** Analysis of drug-resistant CTCs in different cancer sub-types.

Tumor type	Drug resistance	Main findings	Ref.
Breast cancer	Actomyosin resistance	CTCs can survive hemodynamic shear stress and chemotherapy thereby enhancing metastases.	(161)
Breast cancer	CTCs having resistance to TRAIL-mediated apoptosis	Studies demonstrate that CTCs survive the TNF cytokine-mediated killing via downregulation of death receptor	(162)
Prostate cancer	CTCs positive for nuclear-localized androgen receptor splice variant 7 (AR-V7) in metastatic castration-resistant	Detection of even one CTC with nuclear AR-V7 positivity translates to resistance to enzalutamide and abiraterone and sensitivity to the taxanes in mCRPC	(163)
Breast cancer	HER2^+^ CTCs resistance to endocrine	Overexpression of the HER2 gene on the surface of CTCs contributes to chemotherapy and endocrine resistance	(155)
Breast cancer	ER+/HER2^-^ CTCs resistance to paclitaxel	The interconverting phenotypes within patient-derived HER2^+^/NOTCH1^-^CTCs and NOTCH inhibitor-sensitive HER2^-^/NOTCH1^+^ CTCs contribute to the progression of breast cancer and acquisition of drug resistance	(100)
Colorectal cancer	DLG7 and BMI-1 expressing CTCs	Orthotopic mice demonstrate an elevated capacity for tumor formation and self-renewal	(164)
Breast cancer	CTCs resistant to resistant to epirubicin and cisplatin	CTCs of breast cancer are more potentiate to DNA repair and acquire resistance	(165)
Prostate cancer	CTCs and DTCs exhibited Mitoxantrone resistance	Comparison between the CTCs and DTCs sensitivity and selective resistance to mitoxantrone that initiate increased cell invasion and metastasis	(166)
Breast cancer	CD133 expressing CTCs	CD133 expressing CTCs demonstrate the potential resistance to systemic chemotherapy and served as a prognostic biomarker	(167)
Colorectal cancer	CEA and CD133 expressing CTCs	Patients with CEA/CD133-positive CTCs demonstrate a poor prognosis and recurrence	(168)
Breast cancer	drug-resistant MRP5 positive-CTCs resistance to cisplatin and carboplatin MRP7 positive CTCs resistance to taxanes CTCs positive for MRP4 MRP5 resistance to methotrexate	Multidrug resistance profile analyzed by expression of MRPs and expression of ALDH1 produce aggressive and resistant CTCs population	(151)
Hormone refractory prostate cancer	IGF-IR-positive CTCs with docetaxel resistance	Detectable IGF-IR expression on CTCs before treatment with figitumumab (CP-751,871) and docetaxel associated with a decrease in prostate-specific antigen	(169)

ALDH1, Aldehyde-dehydrogenase 1; BMI-1, B cell-specific Moloney murine leukemia virus integration site 1; DTCs, disseminated tumor cells; DLG7, discs large homolog 7 (DLG7); IGF-IR, insulin-like growth factor-I Receptor.

Cancer metastasis arises at an early as well as late stage of the disease. Detection of cancer at advanced stages remains mostly incurable and is a foremost cause of death in patients (72). The secondary location for tumor formation in specific organs during metastasis entirely depends on the intrinsic traits of circulating tumor cells and tumor microenvironment (73, 74). The presence and characteristics of CTCs can provide important insights into metastatic progression and treatment response, making them a key focus in cancer research and monitoring. Metastatic CTCs display the fast growth of tumor cells, and colony formation abilities and contribute to disease progression (73). In modern oncology research, the investigation of CTCs biology as a biomarker is decisively significant for the potential diagnosis and therapy (75, 76). Hence, both CTCs and CTC clusters have clinical relevance as prognostic markers in cancer detection and diagnosis (77, 78). Donato et al. have revealed that intra-tumor hypoxia directs the formation of CTCs-clusters with elevated metastatic abilities (79). Additionally, Aceto et al. highlight that the CTCs-clusters have higher metastatic potential than single CTCs. Single-cell RNAseq information of CTC-clusters in patients of breast cancer revealed highly expressed plakoglobin (a cell junction component). The knockdown of plakoglobin in the mouse model inhibits the CTC-cluster formation and suppresses lung metastasis (80). Further, the number of CTCs is usually a predictive biomarker of progression-free survival and overall survival to monitor treatment efficacy (81). Drug resistance profiles on CTCs from epithelial cancer can be potentially used for the predictive marker of resistance to chemotherapy at different stages of the disease. Budd et al. demonstrated the clinical importance and molecular characteristics of CTCs and CTC-clusters in patients with small cell lung cancers (SCLC) who were undergoing standard cisplatin chemotherapy. The subpopulation of proliferating and apoptotic CTCs was found in the blood of patients whereas CTC-clusters develop drug resistance and protection from anoikis (82). Apart from the predictive and prognostic biomarkers CTCs potentially provide information regarding the developed drug resistance during cancer progression. Budd et al. have demonstrated that the number of CTCs is directly correlated with the tumor burden and is best suited to correlate with the overall survival of patients with metastatic breast cancer (83). The development of advanced techniques that diagnose cancer more sensitively helps to avoid delays in cancer diagnosis. Clinical trials reveal the translational significance of CTCs for the response to therapy and treatment. Many clinical trials were conducted where CTCs counts were used as an indicator for disease progression and overall survival. **Table 3** illustrates the brief details of different clinical studies carried out in cancer subtypes by using CTCs for the early prognosis of the disease.

**Table 3:** Prognostic significance of CTCs in clinical trials in cancer subtypes.

Tumor type	Clinical trial No. and Status	CTCs application	Main finding	Ref.
Breast Cancer	NCT00601900, Ongoing	Prognostic potency and therapeutic response monitoring	EpCAM-based detection of ≥5 CTCs in 31% of ER+ metastatic breast cancer (mBC) patients	(170)
	NCT00382018, Completed	Prognostic potency	EpCAM-based immunomagnetic detection of CTCs in metastatic breast cancer (mBC) patients with single-agent 1^st^ line chemotherapy	(171)
	NCT02101385, Ongoing	Prognostic potency	EpCAM-based detection of early TNBC patients for positive CTCs and CtDNA after neoadjuvant chemotherapy independently associated with disease reoccurrence	(172)
	NCT01745757, Completed	Prognostic potency	EpCAM-based detection CTC count ≥5 in 37% of the HER2-negative metastatic breast cancer (mBC) patients.	(173)
	NCT00785291, Completed	Prognostic potency, Guiding strategy	EpCAM-based detection of trajectory CTCs in metastatic breast cancer (mBC) patients during chemotherapy. Intermediate and high CTCs trajectory associated with poor prognosis in mBC patents	(174)
	NCT01701050	Prognostic potency	EpCAM-based detection of CTCs in 36% of the ER+/HER- metastatic breast cancer (mBC) patients. Elevated CTCs count	(175)
Colorectal cancer	NCT01640405, NCT01640444	Prognostic potency	EpCAM-based detection of ≥3 CTCs in 41% metastatic Colorectal cancer (mCRC) patients	(176)
	NCT01442935	Prognostic potency	EpCAM-based detection ≥3 CTCs in the 19% metastatic Colorectal cancer (mCRC) patients four weeks after therapy	(177)
Pancreatic Cancer	NCT01919151	Prognostic value and used for guiding therapy	EpCAm-based detection of CTCs in preoperative patients revealed the detrimental outcomes	(178)
Prostate Cancer	NCT01942837, NCT02025010	Prognostic potency	EpCAM-based detection of CTCs transcriptional profile in metastatic castration-resistant prostate cancer (mCRPC) patients	(179)

A higher count of CTCs in the blood is associated with high tumor burden, poor survival, and cancer relapse (84). Effective chemotherapeutic treatment is often reflected in the reduction of CTC numbers and improved overall survival for patients (85). Additionally, CTCs levels aid clinicians in evaluating the appropriateness of ongoing chemotherapy and assessing the pharmacodynamics of the prescribed drugs (86). Interestingly, studies have also emphasized that the circadian rhythms also dictate the CTC’s dynamic temporal intravasation events and were found elevated during nighttime in mice and patients with breast cancer during rhythmic variation of melatonin levels (87, 88). The studies highlight liquid biopsy sample collection during time-controlled trials exploring their prognostic and predictive potentials. Moreover, the ABC transporters expressed in the CTCs assist them in expelling the toxin substances and drugs from the cells and achieve survival and resistance to apoptosis in circulation (89, 90).

## 7 Role of ABC transporters as CTCs biomarkers

**Figure 1:**
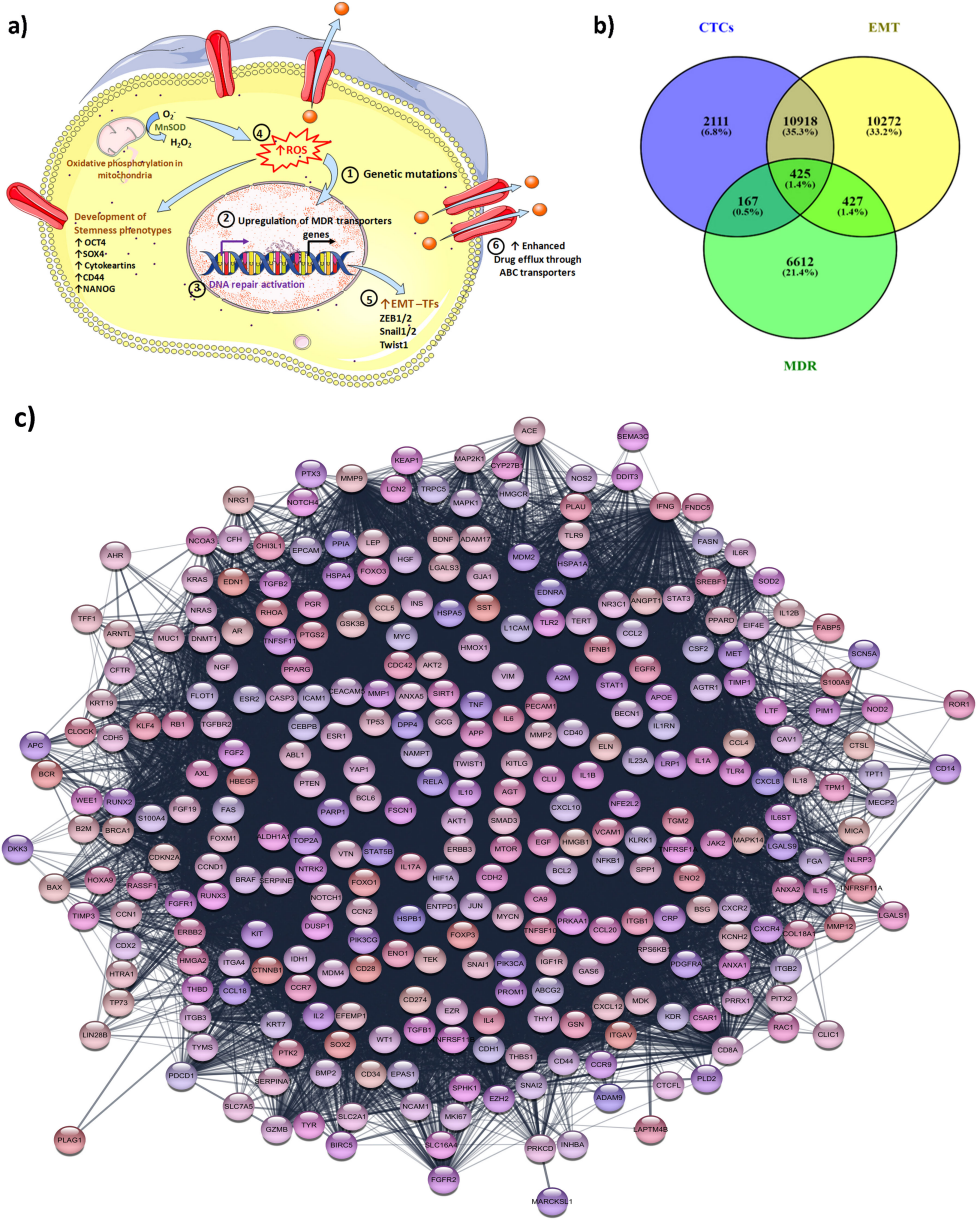
Biological mechanisms responsible for the MDR phenotype in CTCs and their inter-relationship analysis via bioinformatic studies. **(a)** Different mechanisms for the development of MDR phenotype in CTCs: The CTCs achieve stemness phenotypes characterized by upregulation of EMT factors (TWIST, cytokeratins, Snail, etc.) and generation of ROS via activation of oxidative phosphorylation. Elevated ROS is also responsible for genetic mutation, and modulation of redox-sensitive kinases that lead to tumor progression. The CTCs also attain the MDR phenotype via the upregulation of drug-resistance transporters that enhance the intracellular drug efflux from the CTCs. The upregulation of DNA repair activation networks such as the ECRR1 gene helps CTCs to survive and attain chemoresistance phenotype. The process involved in CTCs to attain MDR phenotypes: 1) The epigenetic mutations are responsible for the MDR phenotype; 2) Upregulation of the MDR gene which results in overexpression of ABC transporters; 3) DNA repair activation also facilitates the cells to attain MDR phenotypes; 4) Elevated ROS due also triggers the cells to achieve and develop stemness characteristic; 5) Different transcription factors (TFs) are involved to achieve EMT plasticity; 6) Overexpressed MDR drug transporters are involved in the efflux of an intracellular drug outside from the cells and CTCs become chemoresistant; **(b)** the Venn diagram illustrates the link between the genes involved in the process of EMT, CTCs generation, and MDR development; **(c)** the illustration highlighting the protein-protein interaction among the common top 424 gene involved in EMT, CTCs and MDR mechanisms. The protein-protein linkages were constructed using the STRING database.

Abnormal activation of cell signaling of some oncogenes causes uncontrolled proliferation, metastasis, drug resistance, and relapse. ABC transporters overexpressed in all drug-resistant tumor cells and actively efflux the chemotherapeutic drugs through activating ATP hydrolysis in the extracellular environment (91). Therefore, ABC transporters (P-gp, MRP, and BCRP) typically contribute to multidrug resistance (92). The EMT process induces drug resistance in cells due to the overexpression of ABC transporters (93). Metastatic cancer cells predominantly demonstrate the EMT phenomenon that contributes greatly to the progression of malignant cells and resistance to chemotherapy (94, 95). Various molecular switches come into play during the EMT process and are responsible for contributing stem-cell-like properties to tumor cells thereby developing drug resistance. **Figure 1a** illustrates the biological mechanism responsible for the MDR phenotype in CTCs.

The EMT-mediated CSCs are associated with drug resistance induced by overexpression of MDR-linked ABC efflux transporters. Qu et al. highlighted a significant correlation between the expression of BCRP and P-gp in residual breast cancer tissues and the EMT mechanism, contributing to MDR. Qu et al. demonstrated in their study, that the expression of breast cancer resistance proteins ABCG1 (P-gp) and ABCG2, as well as EMT markers, was assessed in residual breast cancer tissue post-chemotherapy. Results showed that ABCG1, ABCG2, and vimentin were significantly upregulated in the residual tissue compared to pre-chemotherapy samples. Furthermore, ABCG1 and ABCG2 correlated positively with vimentin and negatively with E-cadherin, both at the mRNA and protein levels. These findings suggest that EMT may play a crucial role in the development of MDR in breast cancer with resistance-associated proteins contributing to this process (96). The drug resistance profile of CTCs from epithelial cancers indicates that specific multidrug resistance-related proteins (MRPs) serve as predictive markers for chemotherapy resistance. For example, Gazzaniga et al. showed that 87% of metastatic colorectal cancer patients exhibited overexpression of survivin and MRP5-positive CTCs. Additionally, RT-PCR analysis of CTCs isolated from patients with colorectal cancer and bladder carcinoma revealed positive results for MRP2, MRP4, MRP5, and MRP7. This analysis allows for the identification of specific chemoresistance profiles, even among patients independent of tumor type and stage of disease (97). Overall, the results described the correlation between the CTCs chemoresistance profile outcome in all patients found positive for CTCs in their blood profiles. Gazzaniga and co-workers in their study involving 30 patients with stage IV of various cancer subtypes (bladder (n=7); breast (n=13); colon (n=5), and non-small lung cancer (n=5)) investigated the chemoresistance profiles of CTCs isolated from peripheral blood providing insights into patient responses to standard therapy. Molecular analysis confirmed the presence of MRP2 (resistance to doxorubicin), MRP4 (resistance to cisplatin), and MRP7 (resistance to taxane) that contribute to the development of chemoresistance (98). Furthermore, Gazzaniga et al. demonstrated that the drug resistance profiles of CTCs could predict chemotherapy responses and guide treatment selection across different cancer subtypes. This extensive study on various MRPs (MRP1, MRP2, MRP4, MRP5, and MRP7) establishes a correlation between genes and outcomes such as disease-free survival (DFS) and progression time over two years, confirming that CTC drug resistance profiles are predictive of chemotherapy responses independent of tumor subtypes or disease stage (97, 99).

Jordan et al. demonstrated elevated levels of HER2-positive CTCs in heavily treated breast cancer patients who produce little or none of the HER2 proteins. These HER2-positive CTCs when cultured in the laboratory showed HER2-positive cells replicated into both HER2-positive and HER2-negative cells. The CTCs transformed and maintained discrete HER2-positive and HER2-negative subpopulations (100). These HER2 subpopulations spontaneously interconvert but have different functions. Moreover, HER2-positive CTCs subpopulations showed fast growth, higher lung metastasis, and unlike HER2-positive breast cancer cells *in vitro*. Further, HER2-positive CTCs did not seem to depend on HER2 to survive and were less responsive to HER2-targeted therapy. The HER2-negative CTCs seem to have a slow growth pattern and also less responsive to common chemotherapies but found treatable with γ-secretase inhibitor (GSI)-targeted therapy that blocks the growth-promoting NOTCH pathway in orthotopic CTC-derived tumor NOD scid gamma (NSG) mice models (100). The clinical trial is also going on the HER2-negative breast cancer that has acquired HER2-positive CTCs for overcoming drug resistance and metastasis (101).

## 8 Role of epithelial-to-mesenchymal transition (EMT)-derived CTCs in MDR

**Figure 2:**
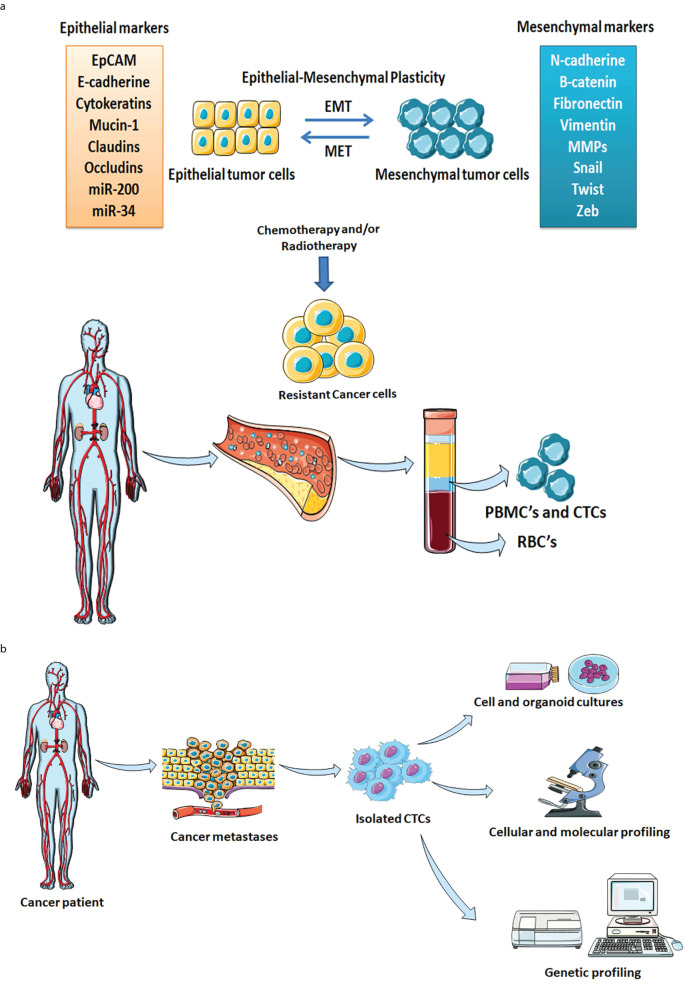
**(a)** Comprehensive representation of dynamic equilibrium between CTCs, drug resistance, and tumor relapse. The EMT plasticity, Cancer heterogeneity, and MDR phenotypes play key roles in the development of chemoresistant CTCs. The chemoresistant CTCs act as seeds for the development of tumors at secondary locations. **(b)** Schematic illustration of CTCs enrichment, analysis, and clinical significance. The isolated CTCs from the cancer patient are used for the single-cell analysis, testing of drug susceptibility, and biological properties of individual tumors. Genetic and molecular profiling facilitates tailoring novel diagnostic and prognostic markers for early cancer detection.

EMT is an important biological process where epithelial cells endure a series of changes that transform them to attain mesenchymal characteristics such as increased migratory and invasive properties. Several studies indicated that during the EMT phenomenon, the transformed cells remain quiescence and generate quiescent CTCs that contribute to cancer metastasis (102, 103). EMT plasticity is a phenomenon of tumor cell transformation between EMT and mesenchymal-to-epithelial transition (MET) plays a crucial role in enabling tumor cells to efficiently migrate from primary tumor to secondary sites (62). The EMT-MET dynamic allows CTCs to adapt and persist in circulation, facilitating metastasis (104). Moreover, the EMT process is associated with the generation of subsets of CTCs, enhanced migration, self-renewal, and drug resistance (105). During the EMT process, the post-transcriptional regulations specifically alternative splicing of CD44 occur and impart a trans-differentiation process that modulates the metastatic potential and chemoresistant properties of cancer cells (106). The EMT process facilitates cancer cells’ survival even after chemotherapy and promotes aggressive lung metastases by enduring tolerance through the activation of chemoresistance-related genes including drug transporters like *ABCB1a, ABCB1b*, and *ABCC1.* Inhibition of EMT has been shown to reduce chemotherapy resistance such as in breast-to-lung metastasis orthotopic mice models where overexpression of mirR-200 reduced cyclophosphamide resistance (107). **Figure 2a** describes the dynamic equilibrium between EMT plasticity, CTCs, and drug resistance. New technologies such as high-throughput sequencing and single-cell sequencing can aid in studying the underlying mechanism of metastases and drug resistance. Single-cell sequencing analysis of CTCs provides insights regarding the genetic and molecular landscapes of tumors and their heterogeneity. The data obtained from the single-cell sequencing reveal that the genotypes and phenotypes of CTCs are inconsistent as compared to the cells present at the primary tumor locations due to resistance developed against given chemotherapy (108). The inconsistency arises from tumor heterogeneity, where CTCs represent only a subset of diverse tumor cells, and from acquired mutations that confer resistance to chemotherapy. Interestingly, the EMT process also alters CTC properties while the blood circulation environment exerts different selective pressure compared to the tumor microenvironment. These factors altogether contribute to the variability in CTC profile thereby complicating their role in cancer progression and treatment outcomes. The identification of metastasis-related markers and therapeutic targets is relevant for the choice of chemotherapy and response. Further, single-cell sequencing analysis explains the EMT phenomena in tumor cells, CTCs, and CTCs clusters in producing metastatic colonies (108). The RNA Sequencing Analysis of CTCs from prostate cancer patients reveals drug resistance by activation of Wnt signaling. The single-cell RNAseq profiles of CTCs from prostate cancer patients (n=13) show evidence of androgen receptor mutations and splicing variants, heterogeneity along with the activation of noncanonical Wnt signaling responsible for the drug resistance (109). The whole genome sequencing of a single CTC isolated from lung cancer patients demonstrated the cancer-associated single nucleotide variations and insertions and/or deletion in exomes of CTCs. The patients with small cell lung cancer revealed distinctly copy number variation (CNV) patterns. The cancer-specific CNV patterns are employed as potential biomarkers for CTC-based cancer diagnosis (110). CTCs that undergo the EMT process exhibit the overexpression of EMT markers such as N-cadherin, ZEB-1/2, vimentin, etc (18). Key EMT-inducing transcription factors such as Twist, Slug, and Snail are present and responsible for the tissue remodeling process in tumors (111). Moreover, overexpression of the EMT signature is accountable for evading apoptosis, and escaping immune surveillance ultimately facilitates acquiring resistance (112–114). The ability of tumor cells to sustain the EMT phenotype is highly diverse in cancer subtypes. Epigenetic modifications such as modification of histone proteins and DNA promoter regions are responsible for the regulation of gene expression (115). Epigenetic regulations such as methylation of certain cytosine residues in DNA and covalent modification of the histone proteins (H3 and H4) of nucleosomes are responsible for the reprogramming of epithelial cells (116–118). Peinado et al. have elucidated the relationship between epithelial-to-mesenchymal transition transcription factors (EMT-TFs) and their binding to DNA, which subsequently directs chromatin configuration through histone modifications. Specifically, EMT-TFs regulate epithelial markers such as E-cadherin, encoded by the CDH1 gene, by activating the EMT process. Histone-modifying enzymes facilitate the repression of the CDH1 promoter, thereby silencing E-cadherin expression (119). As the mesenchymal features epigenetically modify and reprogram the epithelial cancer cells to acclimatize in the tumor microenvironment and attain drug resistance. CTCs are highly heterogeneous and are broadly categorized as epithelial CTCs, transitional CTCs (EMT-CTCs), and mesenchymal CTCs. The EMT-CTCs are the CTCs that are in the process of transitioning from epithelial to mesenchymal state (15). Epithelial and EMT-CTCs are primarily associated with the metastasis process during cancer progression, acquiring drug resistance, and relapse (120). Drug resistance and lack of curative treatments in conventional chemotherapy might overlook CTCs and hence a chemoresistant subpopulation develops within patients. This developed chemoresistant subpopulation of CTCs is the most aggressive form and has a genetic similarity with CSCs. Due to their resemblance with CSCs, these CTCs subpopulations allow tumor growth and proficiently evade apoptosis. Studies have confirmed that due to the development of MDR in breast, lung, colorectal, and prostate cancer patients early release of CTCs has been observed (121, 122). The epithelial-origin CTCs widely express EpCAM and cytokeratin markers on their surfaces and are detected in different subtypes of solid tumors *i.e.* breast, liver, colorectal, prostate, pancreatic, head and neck, lung, and endometrial cancer (123). Studies have demonstrated that with disease progression, the expression of EMT markers also increases in CTCs (124–128). Besides EpCAM, vimentin is also found predominately in EMT-positive CTCs. Vimentin marker has superior sensitivity and specificity as compared to EpCAM-based detection during cancer progression and treatment in patients (129). Typically transitional EMT-CTCs population attributed to cancer progression, drug resistance, dormancy, or all mentioned phenotypes. Since CTCs distribution mainly happens through the blood and interestingly only the metastasis-competent population of CTCs can generate metastasis during disease progression (130, 131). Therefore, these CTCs could serve as potent prognostic markers to monitor cancer disease progression, first-line chemotherapy clinical outcomes of patients, drug resistance, and cancer relapse. **Figure 2b** represents the significance of enriched CTCs in drug testing, and molecular and genetic analysis for better chemotherapeutics for patients.

## 9 Role of cancer heterogeneity in CTCs and MDR

The heterogeneity within the tumor with different phenotypes and self-renewal capacity is a crucial factor for multidrug resistance in cancer. The chemotherapy was found to be successful for cell clones within the tumor mass but often demonstrated different effects on low proliferative tumor-initiating cells or cancer stem cells (CSCs) (132, 133). Tumor genetic mutations, transcriptomics, phenotypic profiles, and epigenetic modifications such as DNA methylation are responsible for tumor heterogeneity. Tumor heterogeneity is significantly involved in therapeutic failures and contributes to drug resistance (134). Hypoxia and crosstalk between tumor and stromal cells within the tumor microenvironment manipulate tumor phenotypes and influence the reprogramming of the tumor microenvironment thereby attaining drug resistance (135). The heterogeneity is attained by variability via intratumor heterogeneity (within an individual tumor) and/or intertumor heterogeneity (across different tumors). Tumor clone subpopulations have diverged epigenetic variabilities, metabolic alterations, cellular morphologies, proliferation rates, metastatic potential, vasculature and eventually developing resistance to given therapy (136). During the early stages of tumor growth exhibits a high metabolic requirement of nutrient uptake and biosynthesis. Tumor cells undergo metabolic reprogramming to succeed in the energy requirement, not only ATP but also NADPH, NADH, and carbon skeletons during cancer progression, metastases, and drug resistance (137). The tumor clones interact with the immune cells, stromal cells, and other tumor cells thereby modulating properties such as metabolism and drug responsiveness. Cancer stemness correlates with intratumoral heterogeneity that also contributes to evading the immune checkpoints. The tumor heterogeneity is also responsible for cancer stemness which generates variability within the tumor and clonal evolution under the influence of drug treatment (138). CSCs have intrinsic resistance to chemotherapy and CTCs originating from the CSCs are crucial to detect in the blood of patients. Interestingly, CTCs of CSCs-origin are contributing vastly to MDR due to overexpression of antiapoptotic and ABC transporters genes. Jin et al. demonstrated the comparison of cancer stem-like cell features isolated from *MRP1* resistance glioblastoma and astrocytoma cells. The result showed *MRP1* upregulation and downregulation of the *MRP3* gene in CSCs whereas in differentiating cells, *MRP3* upregulation was observed (139). CTCs are heterogeneous conferring spatial heterogeneity and immune evasion mechanisms according to their spatial vascular sites. Sun et al. investigated the single cell transcriptome analysis from four different vascular sites including the hepatic vein, peripheral artery, peripheral vein, and portal vein in hepatocellular carcinoma patients. The single-cell RNA-seq (ScRNA-Seq) data and spatiotemporal analysis demonstrated a significant heterogeneous nature of CTCs depending on disseminated intravascular and intervascular sites (140). The CSCs-originated CTCs exhibit stem cell-like abilities such as self-renewal, heterogeneity, and clonal expansion that further initiate tumors. CSCs-specific biomarkers CD133 and ABCG2 pumps are expressed in EpCAM-positive CTCs. CTCs with stem-like phenotypes are more aggressive forms of tumors and contribute to early cancer recurrence in patients. Enhanced metastasis and development of tumor relapse occur due to the elevated levels of CTCs and their transformation into CSCs (141). Budd et al. have shown that the number of CTCs is best suited to correlate with the overall survival of patients (83). EMT-positive CTCs transform into relapse-initiating CSCs that further contribute to the enhancement of metastasis and drug resistance. The secondary location of tumor recurrence is influenced by intracellular microenvironmental factors which are adaptive and ambient conditions for relapsed tumor cells thereby promoting cancer progression and tissue-dependent dormancy. Mego et al. demonstrated that a higher level of EMT-transformed CTCs is produced during chemo- and radio-resistance (142). CTCs subclones can be used to induce the phenotypes of CSCs and enable the CTCs to survive in peripheral circulation. The relationship between the EMT, CTCs, CSCs, and dormancy could be used for clinically relevant chemotherapy. Papadaki and co-workers demonstrated the prognostic relevance and effect of chemotherapy on CTCs co-expressing the phenotype of CSCs and partial-EMT in metastatic breast cancer patients on the first line of chemotherapy. The investigation with CSCp/partial-EMTp CTCs in metastatic breast cancer showed a chemoresistant subpopulation and efficient targeting of CSCp/partial-EMT CTCs could potentially increase patient survival (143). A major obstacle in cancer management is due to the heterogeneity of tumors in patients and residual drug resistance developed in tumor cells. The EMT-transformed CTCs also indicate the transition of tumors from dormancy to relapse in patients. Conventional chemotherapy is found unsuccessful in eliminating the tumor cells with CSC and EMT phenotypes that eventually lead to cancer relapse (144). Different research has indicated that the tumor cells stay in the partial state of epithelial and mesenchymal phenotypes along with stemness-like properties that contribute to aggressive tumorigenicity, metastasis, and resistance (95, 143, 145–147). Recent advancements in the area of biomedicine and translational research aid in the suppression of CTCs to prevent the relapse of cancer in patients eventually extending their lifespan.

## 10 Relationship between EMT and CTCs in the development of MDR

The events occurring during EMT such as the downregulation of cytokeratins, loss of epithelial-specific markers, and upregulation of mesenchymal markers including N-cadherin, vimentin, and resistance to apoptosis make the CTCs resilient in circulation. Studies have established the link between EMT and drug resistance particularly multidrug resistance phenotype due to the enhancement of cancer survival and upregulation of drug resistance genes such as drug transporters (*ABCB1A, ABCB1B, and ABCC1*), cytochrome P450s, and aldehyde dehydrogenase (ALDHs) (95, 107). CTCs express the genes for both epithelial and mesenchymal markers. The CTCs showed differences in the EMT gene and MDR gene expression during the metastases of cancer. The bioinformatics analysis was carried out by considering the genes involved in EMT, MDR, and CTCs in the human gene database. The Venn diagram was constructed by using the software from BioInfoGP (Venny 2.1.0 software). The analysis data revealed 424 common genes that are found linked between the EMT, CTCs, and MDR (**Figure 1b**). These 424 genes are essential elements that play an important role in cancer biology and their role contributes to each process. These 424 genes are identified as highly related and interlinked among EMT, CTCs, and MDR demonstrated by the bprotein-protein interaction network of linkage between 424 genes and their association partners in STRING databases (https://string-db.org) (**Figure 1c**). The details of all 424 genes are given in the **Supplementary Table** (**Supplementary Table S1**). Certain EMT transcription factors including TWIST1, SNAIL, and SLUG are critical regulators of EMT that suppress E-cadherin (epithelial marker) and upregulate N-cadherin and vimentin (mesenchymal markers). As the gene promotes EMT thereby enabling circulating tumor cells to enter into the bloodstream. Moreover, EMT transcription factors also induce drug resistance by altering cell differentiation and survival pathways. Liu et al. have demonstrated that the expression of Twist1, an EMT transcriptional factor significantly increased in the vincristine-resistant HCT-8 cells and subsequently chemoresistance by increasing the expression and promoter activities of ABCB1 and ABCC1 in colon cancer cells (148). The EMT-transcriptional factors Snail and Snug are associated with the development of drug resistance by providing p53-mediated resistance to apoptosis (149). Other transcription factors ZEB1 and ZEB2 are responsible for the downregulation of epithelial markers further promoting mesenchymal traits and facilitating the formation of CTCs. ZEB1 and ZEB2 transcription factors alter the survival pathways and inhibit the apoptotic signals thereby contributing to drug resistance.

Vimentin the mesenchymal marker expressed during EMT and its expression is associated with enhanced migratory and invasive capabilities enabling the dislodging of CTCs from the primary tumor into the bloodstream. Vimentin expression is linked with enhanced cellular plasticity and adaptability thereby contributing to drug resistance. Transcription factor 4 (TCF4) overexpression promotes doxorubicin resistance and CSC markers such as CD133 in CRC cells. TCF4 promoted the overexpression of ZEB1 and ZEB2 leading to proliferation and doxorubicin resistance in CRC cells (150). The ABC transporters (ABCB1, ABCG2, and ABCC1) expression in CTCs assists cell survival in the bloodstream by expelling toxic substances and drugs from CTCs. ABC transporters are associated directly with MDR development by reducing intracellular concentrations of chemotherapeutic agents making cells less susceptible to chemotherapeutics. The expression of ABC transporters on CTCs is demonstrated to have a response to chemotherapy in breast cancer patients. Gradilone et al. emphasized that patients with MRP-positive CTCs have poor progression-free survival and drug resistance (151). The CD44; cancer stem cell markers are involved in cell-cell interactions, cell adhesion, and migration and are often found upregulated in CTCs. The upregulated CD44 in CTCs contributes to their ability to travel through the bloodstream and adhere to distant tissues. Moreover, CD44 expression influences the cell survival pathways thereby contributing massively to drug resistance. CTCs are positive for CD44 and CD74 expression strongly correlated with enhanced plasticity and survival thereby demonstrating drug resistance (152). Understanding these common genes and their roles provides insights into potential therapeutic targets. Moreover, inhibiting these common genes or their pathways facilitates the prevention of metastasis, reduces the formation of CTCs, and overcomes drug resistance leading to the development of more effective and personalized cancer therapy.

## 11 Conclusions and future perspectives

Circulating tumor cells are majorly responsible for metastasis, disease progression, and chemoresistance in patients. EMT phenomenon is accountable for the aggressive nature of CTCs thereby speeding up the metastasis and invasion at secondary locations. During chemotherapy, the dynamic nature of CTCs changes due to the action of drugs. CTCs acquire expression of different genes such as HER2, CD133, CD44, and MRP, etc that are liable for the development of drug resistance and involved in disease progression in patients. The monitoring of CTCs after and during the course of chemotherapy serves as a potential prognostic marker that is connected with chemoresistance and disease progression. Monitoring of CTCs and CTCs-clusters elucidates their biological traits, response to therapy, and drug resistance. Moreover, CTCs significantly focus and highlight the intrapatient tumor heterogeneity and comprehensive insights as compared with the bulk tumor counterparts. Recently, liquid biopsies have facilitated the early detection of potential tumor signatures in bodily fluids. As the amount of tumor material present in biological fluids is very limited, it is crucial to use highly sensitive methodologies to avoid false-negative results and guarantee the best treatment options for patients. Therefore, liquid biopsies have clinical advantages in detecting non-invasive tumor profiling additionally they also facilitate the monitoring of disease onset in asymptomatic individuals. Moreover, a subset of patients with CTCs significantly identifies a compatible profile with specific drug resistance that potentially predicts the disease recurrence and relapse.

The accuracy of CTCs detection proves to be critical in the early detection, diagnosis as well and prognosis of cancers in patients. Moreover, the employment of multiple biomarkers for the isolation and detection of CTCs is the better strategy for enriching all circulating tumor cells that have transformed from either the EMT or MET phase. Moreover, the bioinformatic analysis provides insights regarding the common gene between EMT, CTCs, and MDR. Researchers can target these genes to inhibit their expression, and metastasis process thus decreasing the formation of CTCs and combating drug resistance in patients. However, low densities of CTCs in peripheral blood samples and bodily fluids can hamper the clinical utility of the traditional approaches to their detection. Thus, novel, sensitive, and cost-effective strategies with the applications of nanomaterials could prove significant in clinical effectiveness in managing drug resistance. The nanomaterials have extraordinary optical and physical properties that amplify the low signals and improve the sensitivity. The liquid biopsies technology is still evolving and has promising opportunities to evaluate drug resistance profiling in clinical oncology.

Despite the advancement in detecting CTCs and their potential as prognostic markers, there is currently a lack of standardized methods for their identification and characterization. The unpredictability of detection methods, biomarker selection, and isolation methods can lead to inconsistent results and hinder the clinical utility of CTCs. The inconsistency poses challenges in assessing tumor heterogeneity accurately, drug resistance, and treatment response. Establishing such standards is crucial for maximizing the potential of CTCs in personalized medicine and improving cancer management outcomes. Therefore, development in the area of CTCs research allows for tailored treatment strategies that utilize the unique characteristics of each patient’s cancer and improve personalized medicine and effective cancer management.
