# Supplementary material for: Circulating tumor cell markers for early detection and drug resistance assessment through liquid biopsy
Source: Front Oncol. 2025 Apr 7;15:1494723. doi: 10.3389/fonc.2025.1494723 (PMC12009936; doi:10.3389/fonc.2025.1494723)
Supplement: Supplementary file 1 [file Table1.docx]

**Circulating Tumor Cell Markers for Early Detection and Drug Resistance Assessment through Liquid Biopsy**

Priya Yadav^1^, Saravanan Rajendrasozhan^2,4^, Ramzi Hadj Lajimi^2,4^, Raja Ramadevi Patel^3,4^, Dominique Heymann^5,6,7^, N. Rajendra Prasad^1*^

^1^Department of Biochemistry and Biotechnology, Annamalai University, Annamalai Nagar, Chidambaram 608002, Tamil Nadu, India

^2^Department of Chemistry, College of Science, University of Ha'il, Ha'il 55473, Saudi Arabia

^3^Department of Biology, College of Science, University of Ha'il, Ha'il 55473, Saudi Arabia

^4^Medical and Diagnostic Research Centre, University of Ha'il, Hail 55473, Saudi Arabia

^5^Nantes Université, CNRS, US2B, UMR 6286, 44300 Nantes, France

^6^Institut de Cancérologie de l’Ouest, Tumor Heterogeneity and Precision Medecine Laboratory, 44805 Saint-Herblain, France

^7^University of Sheffield, Medical School, S10 2RX Sheffield, UK

**Table S1: Table illustrates the top 424 common genes interlinked between EMT mechanism, generation of CTCs, and development of MDR**

| **S. No.** | **Gene symbol** | **Gene description** |
| --- | --- | --- |
|  | TP53 | Tumor protein p53 |
|  | TNF | Tumor necrosis factor |
|  | TNFRSF11B | TNF receptor superfamily member 11b |
|  | CDKN2A | Cyclin dependent kinase inhibitor 2A |
|  | TNFRSF1A | TNF receptor superfamily member 1A |
|  | CXCL8 | C-X-C motif chemokine ligand 8 |
|  | FAS | Fas cell surface death receptor |
|  | NFKB1 | Nuclear factor kappa B subunit 1 |
|  | WT1 | WT1 transcription factor |
|  | TNFSF11 | TNF superfamily member 11 |
|  | TNFSF10 | TNF superfamily member 10 |
|  | RELA | RELA proto-oncogene, NF-kB subunit |
|  | MUC1 | Mucin 1, cell surface associated |
|  | APOE | Apolipoprotein E |
|  | HGF | Hepatocyte growth factor |
|  | ADAM17 | ADAM metallopeptidase domain 17 |
|  | PTEN | Phosphatase and tensin homolog |
|  | APC | APC regulator of WNT signaling pathway |
|  | PTX3 | Pentraxin 3 |
|  | TP73 | Tumor protein p73 |
|  | EPCAM | Epithelial cell adhesion molecule |
|  | TNFRSF11A | TNF receptor superfamily member 11a |
|  | BSG | Basigin (Ok blood group) |
|  | SERPINE1 | Serpin family E member 1 |
|  | VEGFA | Vascular endothelial growth factor A |
|  | IL6 | Interleukin 6 |
|  | FOXM1 | Forkhead box M1 |
|  | RASSF1 | Ras association domain family member 1 |
|  | NLRP3 | NLR family pyrin domain containing 3 |
|  | KRAS | KRAS proto-oncogene, GTPase |
|  | LGALS9 | Galectin 9 |
|  | KIT | KIT proto-oncogene, receptor tyrosine kinase |
|  | CRP | C-reactive protein |
|  | AR | Androgen receptor |
|  | TPT1 | Tumor protein, translationally-controlled 1 |
|  | PPARG | Peroxisome proliferator activated receptor gamma |
|  | TGFB1 | Transforming growth factor beta 1 |
|  | LGALS3 | Galectin 3 binding protein |
|  | EGFR | Epidermal growth factor receptor |
|  | CD274 | CD274 molecule |
|  | TYR | Tyrosinase |
|  | CDH1 | Cadherin 1 |
|  | TLR4 | Toll like receptor 4 |
|  | IL10 | Interleukin 10 |
|  | IFNG | Interferon gamma |
|  | JAK2 | Janus kinase 2 |
|  | MDM2 | MDM2 proto-oncogene |
|  | ITGB3 | Integrin subunit beta 3 |
|  | STAT3 | Signal transducer and activator of transcription 3 |
|  | ERBB2 | erb-b2 receptor tyrosine kinase 2 |
|  | IL1RN | Interleukin 1 receptor antagonist |
|  | AKT1 | AKT serine/threonine kinase 1 |
|  | ESR1 | Estrogen receptor 1 |
|  | BRCA1 | BRCA1 DNA repair associated |
|  | HMGB1 | High mobility group box 1 |
|  | ACE | Angiotensin I converting enzyme |
|  | BDNF | Brain derived neurotrophic factor |
|  | BRAF | B-Raf proto-oncogene, serine/threonine kinase |
|  | MMP9 | Matrix metallopeptidase 9 |
|  | LEP | Leptin |
|  | FOXO3 | Forkhead box O3 |
|  | KITLG | KIT ligand |
|  | RB1 | RB transcriptional corepressor 1 |
|  | IL17A | Interleukin 17A |
|  | HIF1A | Hypoxia inducible factor 1 subunit alpha |
|  | CD44 | CD44 molecule (In blood group) |
|  | CCL2 | C-C motif chemokine ligand 2 |
|  | PTGS2 | Prostaglandin-endoperoxide synthase 2 |
|  | IL1B | Interleukin 1 beta |
|  | TERT | Telomerase reverse transcriptase |
|  | VDR | Vitamin D receptor |
|  | CXCR4 | C-X-C motif chemokine receptor 4 |
|  | FOXP3 | Forkhead box P3 |
|  | PDCD1 | Programmed cell death 1 |
|  | MMP2 | Matrix metallopeptidase 2 |
|  | CFH | Complement factor H |
|  | APP | Amyloid beta precursor protein |
|  | MIR21 | MicroRNA 21 |
|  | PIK3CA | Phosphatidylinositol-4,5-bisphosphate 3-kinase catalytic subunit alpha |
|  | IL6R | Interleukin 6 receptor |
|  | EDN1 | Endothelin 1 |
|  | H19 | H19 imprinted maternally expressed transcript |
|  | CHI3L1 | Chitinase 3 like 1 |
|  | CXCL12 | C-X-C motif chemokine ligand 12 |
|  | SPP1 | Secreted phosphoprotein 1 |
|  | IL18 | Interleukin 18 |
|  | SIRT1 | Sirtuin 1 |
|  | ICAM1 | Intercellular adhesion molecule 1 |
|  | MTOR | Mechanistic target of rapamycin kinase |
|  | BCL2 | BCL2 apoptosis regulator |
|  | TGFBR2 | Transforming growth factor beta receptor 2 |
|  | THBS1 | Thrombospondin 1 |
|  | MYCN | MYCN proto-oncogene, bHLH transcription factor |
|  | NOTCH1 | Notch receptor 1 |
|  | TLR2 | Toll like receptor 2 |
|  | CTNNB1 | Catenin beta 1 |
|  | LCN2 | Lipocalin 2 |
|  | NOD2 | Nucleotide binding oligomerization domain containing 2 |
|  | MIR146A | MicroRNA 146a |
|  | NFE2L2 | NFE2 like bZIP transcription factor 2 |
|  | NAMPT | Nicotinamide phosphoribosyltransferase |
|  | MIR155 | MicroRNA 155 |
|  | CD14 | CD14 molecule |
|  | MAPK1 | Mitogen-activated protein kinase 1 |
|  | MYC | MYC proto-oncogene, bHLH transcription factor |
|  | MIR34A | MicroRNA 34a |
|  | IL4 | Interleukin 4 |
|  | CXCL10 | C-X-C motif chemokine ligand 10 |
|  | VCAM1 | Vascular cell adhesion molecule 1 |
|  | BIRC5 | Baculoviral IAP repeat containing 5 |
|  | MET | MET proto-oncogene, receptor tyrosine kinase |
|  | STAT1 | Signal transducer and activator of transcription 1 |
|  | MICA | MHC class I polypeptide-related sequence A |
|  | CD40 | CD40 molecule |
|  | KDR | Kinase insert domain receptor |
|  | TIMP1 | TIMP metallopeptidase inhibitor 1 |
|  | DPP4 | Dipeptidyl peptidase 4 |
|  | CLU | Clusterin |
|  | ITGB1 | Integrin subunit beta 1 |
|  | MIR145 | MicroRNA 145 |
|  | AGT | Angiotensinogen |
|  | ESR2 | Estrogen receptor 2 |
|  | TWIST1 | Twist family bHLH transcription factor 1 |
|  | MAPK14 | Mitogen-activated protein kinase 14 |
|  | IGF1R | Insulin like growth factor 1 receptor |
|  | CCND1 | Cyclin D1 |
|  | HMOX1 | Heme oxygenase 1 |
|  | S100A9 | S100 calcium binding protein A9 |
|  | IL12B | Interleukin 12B |
|  | MIR29A | MicroRNA 29a |
|  | CASP3 | Caspase 3 |
|  | HSPB1 | Heat shock protein family B (small) member 1 |
|  | TLR9 | Toll like receptor 9 |
|  | EZH2 | Enhancer of zeste 2 polycomb repressive complex 2 subunit |
|  | LRP1 | LDL receptor related protein 1 |
|  | PARP1 | Poly(ADP-ribose) polymerase 1 |
|  | MMP1 | Matrix metallopeptidase 1 |
|  | CCL5 | C-C motif chemokine ligand 5 |
|  | NOS2 | Nitric oxide synthase 2 |
|  | BAX | BCL2 associated X, apoptosis regulator |
|  | RHOA | Ras homolog family member A |
|  | AHR | Aryl hydrocarbon receptor |
|  | MKI67 | Marker of proliferation Ki-67 |
|  | YAP1 | Yes1 associated transcriptional regulator |
|  | BMAL1 | Basic helix-loop-helix ARNT like 1 |
|  | MALAT1 | Metastasis associated lung adenocarcinoma transcript 1 |
|  | MIR221 | MicroRNA 221 |
|  | PROM1 | Prominin 1 |
|  | MIR126 | MicroRNA 126 |
|  | GSK3B | Glycogen synthase kinase 3 beta |
|  | IL1A | Interleukin 1 alpha |
|  | FGF2 | Fibroblast growth factor 2 |
|  | CFTR | CF transmembrane conductance regulator |
|  | IDH1 | Isocitrate dehydrogenase (NADP(^+^)) 1 |
|  | LTF | Lactotransferrin |
|  | CAV1 | Caveolin 1 |
|  | HSPA5 | Heat shock protein family A (Hsp70) member 5 |
|  | SOD2 | Superoxide dismutase 2 |
|  | NRG1 | Neuregulin 1 |
|  | NR3C1 | Nuclear receptor subfamily 3 group C member 1 |
|  | CD34 | CD34 molecule |
|  | ABCG2 | ATP binding cassette subfamily G member 2 (JR blood group) |
|  | PTK2 | Protein tyrosine kinase 2 |
|  | RAC1 | Rac family small GTPase 1 |
|  | MIR223 | MicroRNA 223 |
|  | IL2 | Interleukin 2 |
|  | JUN | Jun proto-oncogene, AP-1 transcription factor subunit |
|  | HOTAIR | HOX transcript antisense RNA |
|  | AGTR1 | Angiotensin II receptor type 1 |
|  | MIR210 | MicroRNA 210 |
|  | VIM | Vimentin |
|  | BMP2 | Bone morphogenetic protein 2 |
|  | MIR143 | MicroRNA 143 |
|  | SLC2A1 | Solute carrier family 2 member 1 |
|  | SERPINA1 | Serpin family A member 1 |
|  | INS | Insulin |
|  | ITGAV | Integrin subunit alpha V |
|  | VTN | Vitronectin |
|  | KRT19 | Keratin 19 |
|  | FGFR2 | Fibroblast growth factor receptor 2 |
|  | EGF | Epidermal growth factor |
|  | GJA1 | Gap junction protein alpha 1 |
|  | IL15 | Interleukin 15 |
|  | FGFR1 | Fibroblast growth factor receptor 1 |
|  | ITGA4 | Integrin subunit alpha 4 |
|  | PGR | Progesterone receptor |
|  | GAS6 | Growth arrest specific 6 |
|  | SOX2 | SRY-box transcription factor 2 |
|  | MIR125A | MicroRNA 125a |
|  | HSPA4 | Heat shock protein family A (Hsp70) member 4 |
|  | ABL1 | ABL proto-oncogene 1, non-receptor tyrosine kinase |
|  | KLF4 | KLF transcription factor 4 |
|  | MIR375 | MicroRNA 375 |
|  | HSPA1A | Heat shock protein family A (Hsp70) member 1A |
|  | NOTCH4 | Notch receptor 4 |
|  | SMAD3 | SMAD family member 3 |
|  | FOXO1 | Forkhead box O1 |
|  | MIR195 | MicroRNA 195 |
|  | DNMT1 | DNA methyltransferase 1 |
|  | NRAS | NRAS proto-oncogene, GTPase |
|  | RUNX2 | RUNX family transcription factor 2 |
|  | SPHK1 | Sphingosine kinase 1 |
|  | LGALS1 | Galectin 1 |
|  | BECN1 | Beclin 1 |
|  | MIR27A | MicroRNA 27a |
|  | CCN2 | Cellular communication network factor 2 |
|  | CDC42 | Cell division cycle 42 |
|  | B2M | Beta-2-microglobulin |
|  | TYMS | Thymidylate synthetase |
|  | MIR125B1 | MicroRNA 125b-1 |
|  | SNAI1 | Snail family transcriptional repressor 1 |
|  | MIR22 | MicroRNA 22 |
|  | CCN1 | Cellular communication network factor 1 |
|  | MIR200C | MicroRNA 200c |
|  | HMGA2 | High mobility group AT-hook 2 |
|  | TGM2 | Transglutaminase 2 |
|  | AXL | AXL receptor tyrosine kinase |
|  | ANXA2 | Annexin A2 |
|  | CXCR2 | C-X-C motif chemokine receptor 2 |
|  | PRKCD | Protein kinase C delta |
|  | EPAS1 | Endothelial PAS domain protein 1 |
|  | CCR7 | C-C motif chemokine receptor 7 |
|  | NCAM1 | Neural cell adhesion molecule 1 |
|  | IL23A | Interleukin 23 subunit alpha |
|  | ALDH1A1 | Aldehyde dehydrogenase 1 family member A1 |
|  | ERBB3 | Erb-b2 receptor tyrosine kinase 3 |
|  | TOP2A | DNA topoisomerase II alpha |
|  | CA9 | Carbonic anhydrase 9 |
|  | ANGPT1 | Angiopoietin 1 |
|  | KLRK1 | Killer cell lectin like receptor K1 |
|  | PLAU | Plasminogen activator, urokinase |
|  | MECP2 | Methyl-CpG binding protein 2 |
|  | KCNH2 | Potassium voltage-gated channel subfamily H member 2 |
|  | CDX2 | Caudal type homeobox 2 |
|  | RUNX3 | RUNX family transcription factor 3 |
|  | EZR | Ezrin |
|  | MIR17 | MicroRNA 17 |
|  | FASN | Fatty acid synthase |
|  | PDGFRA | Platelet derived growth factor receptor alpha |
|  | CDH2 | Cadherin 2 |
|  | PRKAA1 | Protein kinase AMP-activated catalytic subunit alpha 1 |
|  | RPS6KB1 | Ribosomal protein S6 kinase B1 |
|  | TIMP3 | TIMP metallopeptidase inhibitor 3 |
|  | PLD2 | Phospholipase D2 |
|  | NEAT1 | Nuclear paraspeckle assembly transcript 1 |
|  | HMGCR | 3-hydroxy-3-methylglutaryl-coA reductase |
|  | MIR130A | MicroRNA 130a |
|  | MIR222 | MicroRNA 222 |
|  | FNDC5 | Fibronectin type III domain containing 5 |
|  | PECAM1 | Platelet and endothelial cell adhesion molecule 1 |
|  | MIR214 | MicroRNA 214 |
|  | ANXA1 | Annexin A1 |
|  | EIF4E | Eukaryotic translation initiation factor 4E |
|  | MIR206 | MicroRNA 206 |
|  | S100A4 | S100 calcium binding protein A4 |
|  | MIR16-1 | MicroRNA 16-1 |
|  | KEAP1 | Kelch like ECH associated protein 1 |
|  | CEBPB | CCAAT enhancer binding protein beta |
|  | TEK | TEK receptor tyrosine kinase |
|  | GAS5 | Growth arrest specific 5 |
|  | BCR | BCR activator of RhoGEF and GTPase |
|  | SNAI2 | Snail family transcriptional repressor 2 |
|  | CEACAM5 | CEA cell adhesion molecule 5 |
|  | SREBF1 | Sterol regulatory element binding transcription factor 1 |
|  | BCL6 | BCL6 transcription repressor |
|  | ITGB2 | Integrin subunit beta 2 |
|  | GCG | Glucagon |
|  | MIR20A | MicroRNA 20a |
|  | MIR140 | MicroRNA 140 |
|  | L1CAM | L1 cell adhesion molecule |
|  | UCA1 | Urothelial cancer associated 1 |
|  | MAP2K1 | Mitogen-activated protein kinase kinase 1 |
|  | TGFB2 | Transforming growth factor beta 2 |
|  | GZMB | Granzyme B |
|  | CDKN2B-AS1 | CDKN2B antisense RNA 1 |
|  | NTRK2 | Neurotrophic receptor tyrosine kinase 2 |
|  | MEG3 | Maternally expressed 3 |
|  | PIK3CG | Phosphatidylinositol-4,5-bisphosphate 3-kinase catalytic subunit gamma |
|  | MIR15A | MicroRNA 15a |
|  | MIR144 | MicroRNA 144 |
|  | CD28 | CD28 molecule |
|  | MIR141 | MicroRNA 141 |
|  | MIR30A | MicroRNA 30a |
|  | MIRLET7A1 | MicroRNA let-7a-1 |
|  | HBEGF | Heparin binding EGF like growth factor |
|  | MIR27B | MicroRNA 27b |
|  | GSN | Gelsolin |
|  | MIR148A | MicroRNA 148a |
|  | SCN5A | Sodium voltage-gated channel alpha subunit 5 |
|  | MIR205 | MicroRNA 205 |
|  | HTRA1 | Htra serine peptidase 1 |
|  | NCOA3 | Nuclear receptor coactivator 3 |
|  | CCL20 | C-C motif chemokine ligand 20 |
|  | MIR451A | MicroRNA 451a |
|  | MIR199A1 | MicroRNA 199a-1 |
|  | MIR149 | MicroRNA 149 |
|  | PIM1 | Pim-1 proto-oncogene, serine/threonine kinase |
|  | FSCN1 | Fascin actin-bundling protein 1 |
|  | NGF | Nerve growth factor |
|  | AKT2 | AKT serine/threonine kinase 2 |
|  | CSF2 | Colony stimulating factor 2 |
|  | ENO1 | Enolase 1 |
|  | MIR1-1 | MicroRNA 1-1 |
|  | IFNB1 | Interferon beta 1 |
|  | MDM4 | MDM4 regulator of p53 |
|  | THBD | Thrombomodulin |
|  | PPIA | Peptidylprolyl isomerase A |
|  | THY1 | Thy-1 cell surface antigen |
|  | PPARD | Peroxisome proliferator activated receptor delta |
|  | MIR181A1 | MicroRNA 181a-1 |
|  | SLC7A5 | Solute carrier family 7 member 5 |
|  | INHBA | Inhibin subunit beta A |
|  | MIR200B | MicroRNA 200b |
|  | STAT5B | Signal transducer and activator of transcription 5B |
|  | DDIT3 | DNA damage inducible transcript 3 |
|  | MIR497 | MicroRNA 497 |
|  | GPX1 | Glutathione peroxidase 1 |
|  | DKK3 | Dickkopf WNT signaling pathway inhibitor 3 |
|  | MIR200A | MicroRNA 200a |
|  | MIR139 | MicroRNA 139 |
|  | CTSL | Cathepsin L |
|  | IL6ST | Interleukin 6 cytokine family signal transducer |
|  | MDK | Midkine |
|  | MMP12 | Matrix metallopeptidase 12 |
|  | MIR133A1 | MicroRNA 133a-1 |
|  | ADAM9 | ADAM metallopeptidase domain 9 |
|  | ANXA5 | Annexin A5 |
|  | ENO2 | Enolase 2 |
|  | CCL18 | C-C motif chemokine ligand 18 |
|  | MIR19A | MicroRNA 19a |
|  | CDH5 | Cadherin 5 |
|  | MIR23A | MicroRNA 23a |
|  | MIR100 | MicroRNA 100 |
|  | WEE1 | WEE1 G2 checkpoint kinase |
|  | CD8A | CD8 subunit alpha |
|  | CLOCK | Clock circadian regulator |
|  | CCL4 | C-C motif chemokine ligand 4 |
|  | MIRLET7B | MicroRNA let-7b |
|  | MIR1246 | MicroRNA 1246 |
|  | C5AR1 | Complement C5a receptor 1 |
|  | FABP5 | Fatty acid binding protein 5 |
|  | MIR133B | MicroRNA 133b |
|  | MIR106B | MicroRNA 106b |
|  | MIR7-1 | MicroRNA 7-1 |
|  | MIR218-1 | MicroRNA 218-1 |
|  | HOXA9 | Homeobox A9 |
|  | MIR34C | MicroRNA 34c |
|  | EFEMP1 | EGF containing fibulin extracellular matrix protein 1 |
|  | MIR335 | MicroRNA 335 |
|  | FGF19 | Fibroblast growth factor 19 |
|  | MIR378A | MicroRNA 378a |
|  | MIR146B | MicroRNA 146b |
|  | EDNRA | Endothelin receptor type A |
|  | ENTPD1 | Ectonucleoside triphosphate diphosphohydrolase 1 |
|  | MIR424 | MicroRNA 424 |
|  | MIR186 | MicroRNA 186 |
|  | MIR494 | MicroRNA 494 |
|  | MIR106A | MicroRNA 106a |
|  | ELN | Elastin |
|  | MIR224 | MicroRNA 224 |
|  | ROR1 | Receptor tyrosine kinase like orphan receptor 1 |
|  | TFF1 | Trefoil factor 1 |
|  | MIR26B | MicroRNA 26b |
|  | MIR134 | MicroRNA 134 |
|  | TPM1 | Tropomyosin 1 |
|  | HULC | Hepatocellular carcinoma up-regulated long non-coding RNA |
|  | PITX2 | Paired like homeodomain 2 |
|  | MIR483 | MicroRNA 483 |
|  | DUSP1 | Dual specificity phosphatase 1 |
|  | MIR15B | MicroRNA 15b |
|  | A2M | Alpha-2-macroglobulin |
|  | MIR23B | MicroRNA 23b |
|  | LAPTM4B | Lysosomal protein transmembrane 4 beta |
|  | MIR320A | MicroRNA 320a |
|  | SNHG16 | Small nucleolar RNA host gene 16 |
|  | KRT7 | Keratin 7 |
|  | CLIC1 | Chloride intracellular channel 1 |
|  | SLC16A4 | Solute carrier family 16 member 4 |
|  | MIR18A | MicroRNA 18a |
|  | MIR455 | MicroRNA 455 |
|  | MIR130B | MicroRNA 130b |
|  | MIR129-1 | MicroRNA 129-1 |
|  | MIR181B1 | MicroRNA 181b-1 |
|  | MIR215 | MicroRNA 215 |
|  | MIR34B | MicroRNA 34b |
|  | CYTOR | Cytoskeleton regulator RNA |
|  | MIR328 | MicroRNA 328 |
|  | MIR211 | MicroRNA 211 |
|  | FLOT1 | Flotillin 1 |
|  | MIR10A | MicroRNA 10a |
|  | CASC2 | Cancer susceptibility 2 |
|  | DANCR | Differentiation antagonizing non-protein coding RNA |
|  | ZFAS1 | ZNFX1 antisense RNA 1 |
|  | MIRLET7C | MicroRNA let-7c |
|  | FGA | Fibrinogen alpha chain |
|  | MIR135A1 | MicroRNA 135a-1 |
|  | MIR19B1 | MicroRNA 19b-1 |
|  | MIR135B | MicroRNA 135b |
|  | MIR216A | MicroRNA 216a |
|  | MIR127 | MicroRNA 127 |
|  | MIR373 | MicroRNA 373 |
|  | MIR218-2 | MicroRNA 218-2 |
|  | MIR363 | MicroRNA 363 |
|  | HNF1A-AS1 | HNF1A antisense RNA 1 |
|  | MIR129-2 | MicroRNA 129-2 |
|  | MIR16-2 | MicroRNA 16-2 |
|  | MIR491 | MicroRNA 491 |
|  | SST | Somatostatin |
|  | MIR199A2 | MicroRNA 199a-2 |
|  | CCR9 | C-C motif chemokine receptor 9 |
|  | TRPC5 | Transient receptor potential cation channel subfamily C member 5 |
|  | MIR199B | MicroRNA 199b |
|  | SEMA3C | Semaphorin 3C |
|  | PLAG1 | PLAG1 zinc finger |
|  | PRRX1 | Paired related homeobox 1 |
|  | MIR103A1 | MicroRNA 103a-1 |
|  | MIR33B | MicroRNA 33b |
|  | MIRLET7G | MicroRNA let-7g |
|  | MIRLET7D | MicroRNA let-7d |
|  | MIR382 | MicroRNA 382 |
|  | MIR331 | MicroRNA 331 |
|  | MIR625 | MicroRNA 625 |
|  | MIRLET7F1 | MicroRNA let-7f-1 |
|  | MARCKSL1 | MARCKS like 1 |
